# Variation in Outcomes Associated With Blunt Splenic Injury Management

**DOI:** 10.7759/cureus.76997

**Published:** 2025-01-06

**Authors:** Richard Bagdonas, Catherine Caronia, Michael W West, Lauren Rothburd, Shafieh Makehei, Blaze Bagdonas, Despina Bubaris, Karen Fitzgerald, Fathia Qandeel, Theresa Drucker, Heather Reens, Sarah Eckardt, Patricia A Eckardt

**Affiliations:** 1 Trauma Surgery, Good Samaritan University Hospital, West Islip, USA; 2 Pediatrics, Good Samaritan University Hospital, West Islip, USA; 3 Trauma Surgery/Critical Care, Good Samaritan University Hospital, West Islip, USA; 4 Trauma, Good Samaritan University Hospital, West Islip, USA; 5 Trauma, Good Samaritan University Hospital/New York Institute of Technology (NYIT) College of Osteopathic Medicine, West Islip/Westbury, USA; 6 Trauma/Emergency Medical Services, Good Samaritan University Hospital, West Islip, USA; 7 Nursing, Good Samaritan University Hospital, West Islip, USA; 8 Quality Management, Critical Care Nursing, and Education, Good Samaritan University Hospital, West Islip, USA; 9 Research, Good Samaritan University Hospital, West Islip, USA; 10 Nursing, Molloy University, Rockville Centre, USA; 11 Data Science, Eckardt & Eckardt Consulting, St. James, USA

**Keywords:** angioembolization, bayesian statistics, blunt splenic injury, non-operative management, operative management

## Abstract

Introduction

The management of blunt splenic injury has evolved to include splenic artery embolization in addition to non-surgical management, and splenic surgery. Though research has been conducted examining outcomes between management approaches, the inferential findings are often limited by single-site study designs and small sample sizes. However, results from large-scale prior studies can inform inference if a non-frequentist (Bayesian) framework is used. Therefore, the purpose of this study was to examine mortality and length of stay associated with blunt splenic injury management using both frequentist and Bayesian methods.

Methods

A total of 56 patients presenting with blunt splenic injury were included in this retrospective, single-center, quantitative study from January 1, 2021 to December 31, 2022 to inform both methodological approaches. Findings from a national retrospective sample of 117,743 patients presenting with blunt splenic injury between 2007 and 2015 were included in the prior distribution for the Bayesian estimates to provide sufficient statistical power and improve internal validity and generalizability of findings.

Results

Mortality rates and hospital mean length of stay were not significantly different between blunt splenic injury management approaches of non-operative management (n=43), surgery (n=7), and splenic artery embolization (n=6) using a frequentist approach (9.3%, 0%, and 0%, P=.52; and 10.8 (15.8), 10.8 (4.7), and 4.6 (1.8), P=.86, respectively). Bayesian 95% highest density interval (HDI) estimates of the likelihood of mortality ([0.02; 0.18], [-6.4^e-23^; 0.3], and [-2.2^e-22^; 0.3]) and hospital mean length of stay ([7.7; 8.3], [11.0; 12.3], and [8.7; 10.2]) provided reduced uncertainty in point and dispersion estimates.

Conclusions

The inclusion of findings from large high-quality studies provides increased certainty in estimates from smaller studies. Posterior estimates can inform predictive models for testing in future studies.

## Introduction

The most common form of splenic trauma is blunt splenic injury (BSI), frequently caused by accidents involving blunt abdominal trauma. The vascular nature of the spleen increases the risk of complications after injury including extensive arterial bleeding resulting in hemoperitoneum or death [1-4]. Previously, splenectomies were frequently performed as the primary management technique for splenic injury. Currently, practitioners aim to salvage the spleen as it has a number of important functions including T-cell proliferation, antibody production, as well as filtration and storage of erythrocytes and platelets [4,5]. In addition, a multitude of complications can result following splenectomy, furthermore increasing hospital length of stay (LOS), intensive care unit (ICU) LOS, and mortality rates, amongst others [6,7]. Therefore, treatment of BSI has shifted from splenectomy as a default to other forms of intervention in order to improve outcomes and mortality following BSI [1,3,4,7,8].

Providers currently use less invasive interventions such as splenic angioembolization (SAE) and non-operative management (NOM) techniques like fluid resuscitation and blood transfusions to manage BSI [1,2,5]. The current standard of practice in managing BSI is NOM. Intervention is determined by providers based on patient hemodynamic stability, splenic injury scale, injury severity score (ISS), shock index, reverse shock index, presence of a blush on computed tomography (CT) upon arrival, as well as the availability of resources within the institution, which may differ greatly based on facility trauma designation [1,7]. If attempts at initial resuscitation are unsuccessful in establishing hemodynamic stability, alternatively, more invasive solutions are considered, including SAE and operative management (OM), like splenectomy. However, there has been conflicting evidence on which intervention yields the best results when comparing NOM, SAE, and OM. OM frequently results in increased LOS, need for more blood transfusions, greater incidence of mechanical ventilation, and complications such as higher readmission rates and mortality [3, 7-9]. In the literature, studies have cited their findings to indicate that OM patients had a significantly higher ICU LOS and rate of in-hospital mortality compared with the other groups [3,9]. However, due to single-site study designs and small sample sizes, inferences from findings are often limited and not generalizable for future predictive modeling.

Results from small sample sizes limit the interpretation of treatment effects due to a lack of precision. Small participant numbers typically correspond to a small number of endpoint events, often referred to as the “zero numerator problem” [10]. Classical or frequentist statistical methods used in these studies yield excessively conservative results [11]. Considering these challenges, alternative design choices that ensure the utilization of all available data on treatment efficacy should be considered to maximize the ability to draw clinically relevant conclusions. Bayesian methods have been used as a framework to investigate interventions in small samples. Bayesian methods provide an intuitive probability that the treatment effect lies in an estimate range with clinical interpretability and provides more precise estimates when studying treatments in small samples [12].

Bayesian analysis provides computational approaches to update one’s beliefs about certain events (such as mortality and LOS associated with BSI management approach, in our case) by incorporating evidence from high-quality prior studies about those events [13,14]. Specifically, Bayesian inference interprets probability as a measure of credibility that one may possess about the occurrence of an event. Historical information about the topic or event is included with the study information to form a more updated probability distribution [15-17]. In the literature, Bayesian methods have been used to enhance analysis and statistical power concerning appropriate sample sizes [18]. This approach follows the intuitive interpretation of interval estimates fixed ranges to which a parameter is known to belong with a prespecified probability, and an ability to assign an actual probability to any hypothesis [13,15]. A large national retrospective sample (n=117,743) of patients presenting with BSI to level I and II US trauma centers between 2007 and 2015 and treated with the same three comparator interventions provided an informative prior distribution for the Bayesian estimates and enhanced the precision of the posterior estimates of treatment effects.

Therefore, the aim of this exploratory study was to examine the variation in outcomes associated with BSI management approaches using a frequentist approach and a Bayesian approach. The results will be used to inform a priori multivariate model to estimate the association of management approach to BSI and adverse outcomes within a nationally representative sample. Additionally, an introductory free Bayesian software program, FirstBayes, was used for the distribution estimates to present the approach of Bayesian analysis to interested novice users.

## Materials and methods

This investigator-initiated cross-sectional retrospective observational descriptive, correlational, and inferential quantitative pilot research study received Institutional Review Board approval on June 6, 2023 at Good Samaritan University Hospital, a suburban academic hospital on Long Island, NY. The likelihood data included all trauma patients treated at this single site American College of Surgeons (ACS) verified level II trauma center (level II at the time of data collection; currently level I) with a diagnosis of BSI registered in the National Trauma Data Base (NTDB) during the sampling frame of January 1, 2021 through December 31, 2022. Those included in the sample were trauma patients 18 years of age and older with a diagnosis of BSI who had only one modality of management approach (NOM, OM, or SAE). Participants excluded were pediatric, BSI adult patients with emergency department deaths on first admission, penetrating injuries, burn diagnosis, and patients with more than one modality of management approach. Additionally, a national retrospective sample (n=117,743) of patients presenting with BSI between 2007 and 2015 informed the prior distribution for the Bayesian estimates.

Data analysis and statistical methods

The analysis was completed using IBM Statistical Package for the Social Sciences (SPSS) Statistics for Windows, version 28 (IBM Corp., Armonk, NY, USA), Stata Statistical Software: Release 17 (StataCorp LLC, College Station, TX, USA), and FirstBayes Version 1.3 for Windows Vista (Tony O’Hagan, Nottinghamshire, UK) for data recoding, transformation, descriptive, inferential analyses. Descriptive statistics were analyzed with point estimates of central tendency and dispersion for the BSI management approach and hypothesized patient characteristics that influence the management approach. Interval and ratio level measures included estimation of mean and median with 95% CI construction. Nominal and ordinal level measures included estimation of proportion with 95% CI construction. Bayesian modeling was used to construct the prior distribution from existing BSI outcome data. Multivariate estimates were constructed to address the primary aim of obtaining estimates of variation in outcomes (mortality, hospital LOS) associated with the management approach (NOM, OM, and SAE) to BSI within this patient population. The distribution of patient characteristics including demographic data, splenic injury grade, ISS, blood transfusion requirements, LOS, readmission, and mortality was compared across intervention groups including NOM, SAE, and OM. For inferential analyses, a two-tailed testing approach was used for the comparison of patient characteristics between NOM, SAE, and OM intervention groups. Independent sample t-tests (t statistic) and one-way analysis of variance (ANOVA) (f statistic) were estimated for continuous variable estimates. Crosstabulation with chi-square estimates (x2 statistic estimates) was conducted for categorical data comparisons (except when the expected count < 5 in a cell, then Fisher’s exact statistic was estimated). Bayesian posterior and predictive distributions were constructed using the national sample as the prior distribution and the single site sample (n = 117,743) as the likelihood distribution.

## Results

**Figure 1:**
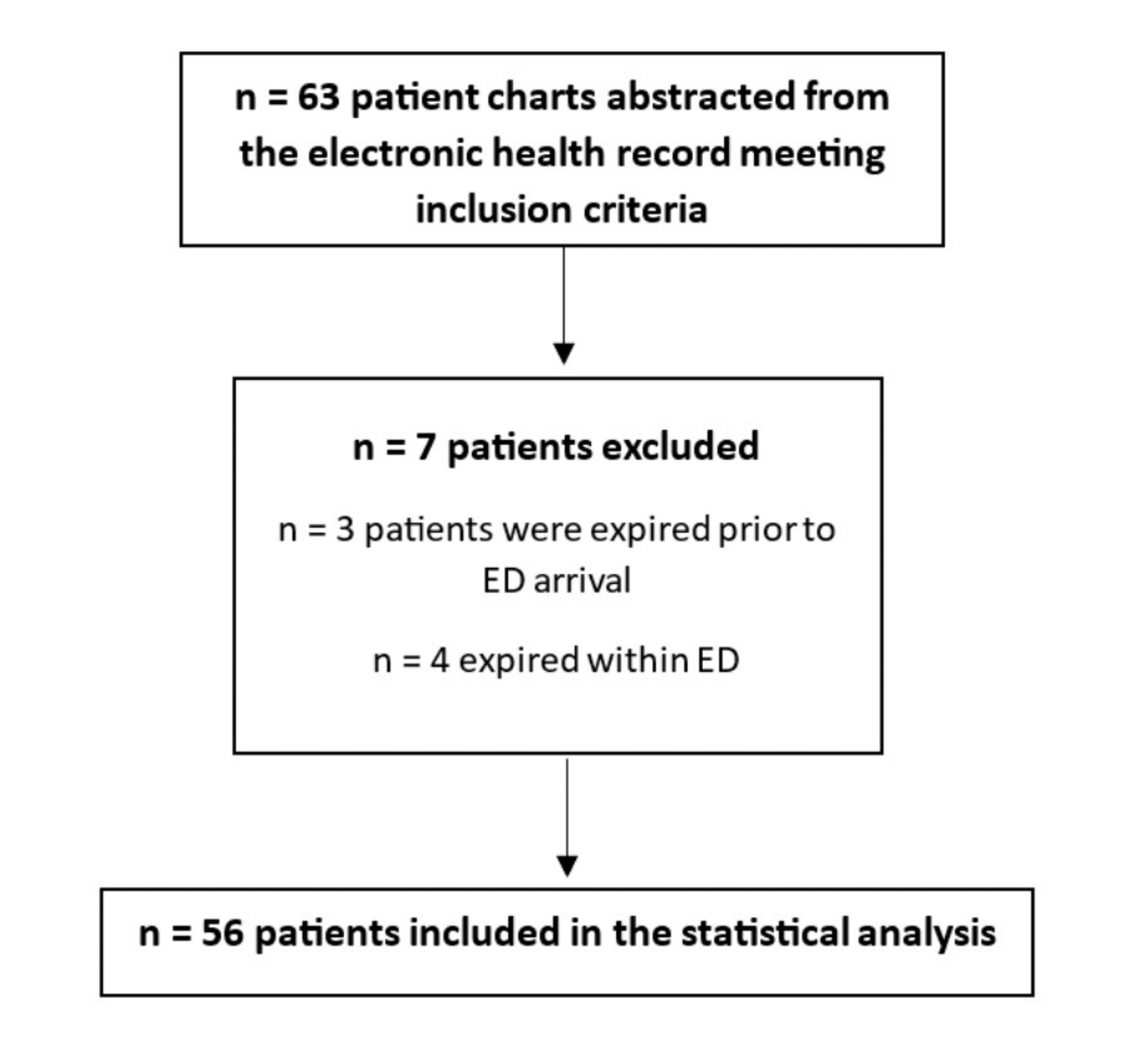
Inclusion Flowchart Likelihood Distribution ED: emergency department

Of the 63 patient records initially extracted from the electronic health record as meeting inclusion criteria for the single-site sample, seven were found ineligible, resulting in 56 patients included in the analysis (Figure 1). Of this total, 43 patients underwent NOM, seven underwent splenectomy (OM), and six patients underwent SAE (Table 1). There were no patients in the sample who underwent splenorrhaphy. Within the prior distribution derived from the national sample, 85,793 patients underwent NOM, 21,999 underwent splenectomy (OM), and 3,895 patients underwent SAE. Mortality rates and hospital mean LOS were not significantly different (Table 2) between BSI management approaches of NOM (n=43), surgery (n=7), and SAE (n=6) using a frequentist approach (9.3%, 0%, and 0%, P = .52) and 10.8 (15.8), 10.8 (4.7), and 4.6 (1.8) P = .86, respectively).

**Table 1:** Descriptive Characteristics of Total Sample (n=56) ^a^Mean, SD. ^b^Frequency, percentage. ED: emergency department; ISS: injury severity score; RR: respiratory rate; BMI: basal metabolic index; INR: international normalized ratio; LOS: length of stay; ICU: intensive care unit; PRBC: packed red blood cells; BSI: blunt splenic injury.

Characteristics	Univariate Statistics
Patient Demographics	
Age^a^	48.7 (2.78)
Sex^b^	
Male	26 (46.4%)
Female	30 (53.6%)
Race^b^	
White	41 (73.2%)
Black	5 (8.9%)
Other	10 (17.9%)
Ethnicity Hispanic/Latino^b^	
Yes	12 (21.4%)
No	44 (78.6%)
Pre-hospital Variables	
Extrication^b^	
Yes	49 (90.7)
No	5 (9.3%)
Time from Injury – Arrival^b^	
< 30 minutes	3 (5.4%)
30-60 minutes	29 (51.8%)
61-90 minutes	0 (0%)
91 minutes-12 hours	5 (8.9%)
>12 hours	17 (30.4%)
Fall^b^	
Yes	26 (46.4%)
No	30 (53.6%)
Fall distance in feet^a^	1.48 (2.66)
ED Variables	
Spleen Grade^b^	
Grade 1	7 (12.5%)
Grade 2	27 (48.2%)
Grade 3	18 (32.1%)
Grade 4	1 (1.8%)
Grade 5	3 (5.4%)
ED Revised Trauma Score^a^	7.60 (0.83)
ED Arrival ISS^a^	14.21 (13.0)
ED Arrival Temperature (F)^a^	97.9 (0.84)
ED Arrival Heart Rate^a^	90.0 (22.22)
ED Arrival RR^a^	19.32 (3.34)
ED RR Assisted^b^	
Yes	2 (3.6%)
No	54 (96.4%)
ED Arrival Systolic Blood Pressure^a^	124.38 (23.52)
BMI^a^	27.8 (6.43)
1^st^ Hematocrit^a^	36.81 (6.81)
1^st^ INR^a^	1.07 (0.11)
ED Shock Index^a^	0.76 (0.30)
ED Reverse Shock Index^a^	1.49 (0.58)
ED High Shock Index^b^	
Yes	12 (21.8%)
No	43 (78.2%)
Number of Comorbidities^a^	1.50 (1.50)
ED LOS (hours)^a^	6.64 (5.50)
ED Disposition^b^	
Floor	8 (14.3%)
Observation	2 (3.6%)
Step Down	6 (10.7%)
ICU	30 (53.6%)
Operating Room	10 (17.9%)
In-hospital Variables	
ICU Admission During Hospital Admission^b^	
Yes	43 (76.8%)
No	13 (23.2%)
ICU LOS (days)^a^	6.95 (12.43)
Ventilator during Hospital Admission^b^	
Yes	10 (22.7%)
No	34 (77.3%)
Admit Service^b^	
Trauma	53 (94.6%)
Internal Medicine	2 (3.6%)
Cardiology	1 (1.8%)
PRBC (0-24 hrs)^b^	
Yes	19 (35.8%)
No	34 (64.2%)
Plasma (0-24 hrs)^b^	
Yes	6 (10.7%)
No	50 (89.3%)
Platelets (0-24 hrs)^b^	
Yes	8 (14.3%)
No	48 (85.7%)
Cryoprecipitate (0-24 hrs)^b^	
Yes	5 (8.9%)
No	51 (91.1%)
VLLA (0-24 hrs)^b^	
Yes	0 (0.0%)
No	56 (100%)
Cellsaver (0-24 hrs)^b^	
Yes	0 (0.0%)
No	56 (100%)
BSI Management^b^	
Non-operative management	43 (76.8%)
Splenectomy	7 (12.5%)
Angioembolization	6 (10.7%)
Outcome Variables	
Hospital LOS (days)^a^	8.16 (11.66)
Readmission^b^	
Yes	5 (8.9%)
No	51 (91.1%)
Readmission LOS (days)^a^	5.60 (2.30)
Unplanned Readmission to Hospital^b^	
Yes	0 (0%)
No	5 (100%)
Mortality^b^	
Yes	4 (7.1%)
No	52 (92.9%)

**Table 2:** Comparison of Variables between NOM, Surgery, and Angioembolization (n=56) ^a^Mean, SD. ^b^Frequency, percentage. ^c^One-way analysis of variance estimates (ANOVA) (f statistic) were conducted for continuous variable. ^d^Crosstabulation chi-square estimates were conducted for categorical data (except when expected count < 5 in a cell Fisher’s exact statistic was estimated). ^e^Inferential statistic calculated. *Significant p value <.05, **Significant p value <.01, ***Significant p value <.001. NOM: non-operative management; ED: emergency department; ISS: injury severity score; RR: respiratory rate; BMI: basal metabolic index; INR: international normalized ratio; LOS: length of stay; ICU: intensive care unit; PRBC: packed red blood cells.

Characteristics	NOM (n=43)	Surgery (n=7)	Angioembolization (n=6)	p-value^c,d^	Test statistics^c,d^
Patient Demographics					
Age^a^	39.6 (16.6)	43.3 (18.84)	52.20 (19.48)	.808^c^	0.214^c^
Sex^b^				.039*^d^	6.514^d^
Male	16 (37.2%)	5 (71.4%)	5 (83.3%)		
Female	27 (62.8%)	2 (28.6%)	1 (16.7%)		
Race^b^				.805^d^	1.622^d^
White	32 (74.4%)	4 (57.1%)	5 (83.3%)		
Black	4 (9.3%)	1 (14.3%)	0 (0.0%)		
Other	7 (16.3%)	2 (28.6%)	1 (16.7%)		
Ethnicity Hispanic/Latino^b^				.297^d^	2.430^d^
Yes	11 (25.6%)	0 (0.0%)	1 (16.7%)		
No	32 (74.4%)	7 (100.0%)	5 (83.3%)		
Pre-hospital Variables					
Extrication^b^				.755^d^	0.283^d^
Yes	5 (11.9%)	0 (0.0%)	0 (0.0%)		
No	37 (88.1%)	7 (100.0%)	6 (100.0%)		
Time from Injury – Arrival^b^				.013*^d^	16.120^d^
< 30 minutes	1 (2.4%)	2 (33.3%)	0 (0.0%)		
30-60 minutes	25 (59.5%)	2 (33.3%)	2 (33.3%)		
61-90 minutes	0 (0.0%)	0 (0.0%)	0 (0.0%)		
91 minutes-12 hours	2 (4.8%)	1 (16.7%)	2 (33.3%)		
>12 hours	14 (33.3%)	1 (16.7%)	2 (33.3%)		
Fall^b^				.109^d^	4.430^d^
Yes	17 (39.5% )	4 (57.15)	5 (83.3%)		
No	26 (60.5%)	3 (42.9%)	1 (16.7%)		
Fall distance in feet^a^	1.47 (2.80)	0.1 (0.05)	2.25 (2.87)	.645^c^	0.450^c^
ED Variables					
Spleen Grade^a^	2.14 (0.71)	3.43 (1.29)	3.0 (1.09)	<.001***^c^	9.482^c^
Spleen Grade^b^				.020*^d^	18.191^d^
Grade 1	7 (16.3%)	0 (0.0%)	0 (0.0%)		
Grade 2	24 (55.8%)	1 (14.3%)	2 (33.3%)		
Grade 3	11 (25.6%)	4 (57.1%)	3 (50.0%)		
Grade 4	1 (2.3%)	0 (0.0%)	0 (0.0%)		
Grade 5	0 (0.0%)	2 (28.6%)	1 (16.7%)		
ED Revised Trauma Score^a^	7.44 (1.60)	7.44 (0.64)	7.70 (0.33)	.932^c^	0.070^c^
ED Arrival Injury Severity Score (ISS)^a^	13.22 (9.13)	11.50 (3.11)	14.50 (6.28)	.208^c^	1.518^c^
ED Arrival Temperature (F)^a^	97.81 (0.79)	98.0 (0.01)	98.0 (1.22)	.957^c^	0.044^c^
ED Arrival Heart Rate^a^	84.33 (23.30)	102.75 (27.37)	97.0 (12.68)	.223^c^	1.544^c^
ED Arrival Respiratory Rate (RR)^a^	18.70 (2.32)	18.50 (1.29)	18 (3.08)	.603^c^	0.511^c^
ED Respiratory Rate Assisted^b^				.179^d^	3.411^d^
Yes	1 (2.3%)	0 (0.0%)	1 (16.7%)		
No	42 (97.7%)	7 (100.0%)	5 (83.3%)		
ED Arrival Systolic Blood Pressure^a^	128.86 (24.03)	113.57 (16.98)	105.67 (12.01)	.030*^c^	3.743^c^
Basal Metabolic Index (BMI)^a^	28.75 (6.47)	23.41 (3.73)	26.21 (4.02)	.133^c^	2.099^c^
1^st^ Hematocrit^a^	36.55 (6.85)	31.76 (9.71)	39.05 (2.89)	.188^c^	1.741^c^
1^st^ INR^a^	1.06 ( 0.07)	1.10 (0.08)	1.00 (0.01)	.557^c^	0.594^c^
ED Shock Index^a^	0.69 (0.24)	0.90 (0.23)	0.92 (0.14)	.096^c^	0.196^c^
ED Reverse Shock Index^a^	1.61 (0.56)	1.16 (0.26)	1.10 (0.15)	.039*^c^	1.078^c^
ED High Shock Index^b^				.049*^d^	6.0777^d^
Yes	7 (16.3%)	3 (42.9%)	3 (50%)		
No	36 (83.7%)	4 (57.1%)	3 (50%)		
Number of Comorbidities^a^	1.37 (1.55)	0.67 (1.03)	2.20 (1.48)	.212^c^	1.597^c^
ED LOS (hours)^a^	6.10 (5.33)	2.95 (1.76)	3.91 (3.25)	.047*^c^	3.249^c^
ED Disposition^b^				.003**^d^	23.454^d^
Floor	8 (18.6%)	0 (0.0%)	0 (0.0%)		
Observation	1 (2.3%)	0 (0.0%)	1 (16.7%)		
Step Down	6 (14.0)	0 (0.0%)	0 (0.0%)		
Intensive Care Unit	25 (58.1%)	2 (28.6%)	3 (50.0%)		
Operating Room	3 (7.0%)	5 (71.4%)	2 (33.3%)		
In-hospital Variables					
ICU Admission During Hospital Admission^b^				.077^d^	5.118^d^
Yes	30 (69.8%)	7 (100.0%)	6 (100.0%)		
No	13 (30.2%)	0 (0.0%)	0 (0.0%)		
ICU LOS (days)^a^	8.11 (15.38)	5.71 (3.15)	5.0 (5.44)	.863^c^	0.148^c^
Ventilator during Hospital Admission^b^				.113^d^	4.360^d^
Yes	7 (16.3%)	3 (42.9%)	0 (0.0%)		
No	36 (83.7%)	4 (57.1%)	6 (100.0%)		
Admit Service^b^				.060^d^	9.043^d^
Trauma	41 (95.3%)	7 (100.0%)	5 (83.3%)		
Internal Medicine	2 (4.7%)	0 (0.0%)	0 (0.0%)		
Cardiology	0 (0.0%)	0 (0.0%)	1 (16.7%)		
PRBC (0-24 hrs)^b^				.001**^d^	14.905^d^
Yes	10 (14.7%)	7 (100.0%)	2 (40.0%)		
No	31 (26.3%)	0 (0.0%)	3 (60.0%)		
Plasma (0-24 hrs)^b^				.012*^d^	8.908^d^
Yes	3 (7.0%)	3 (42.9%)	0 (0.0%)		
No	40 (93.0%)	4 (57.1%)	6 (100.0%)		
Platelets (0-24 hrs)^b^				.002**^d^	12.372^d^
Yes	4 (9.3%)	4 (57.1%)	0 (0.0%)		
No	39 (90.7%)	3 (42.9%)	6 (100.0%)		
Cryo (0-24 hrs)^b^				.128^d^	0.128^d^
Yes	3 (7.0%)	2 (28.6%)	0 (0.0%)		
No	40 (93.0%)	5 (71.4%)	6 (100.0%)		
VLLA (0-24 hrs)^b^				--^d^	NA^d^
Yes	0 (0.0%)	0 (0.0%)	0 (0.0%)		
No	43 (100.0%)	7 (100.0%)	6 (100.0%)		
Cellsaver (0-24 hrs)^b^				--^d^	NA^d^
Yes	0 (0.0%)	0 (0.0%)	0 (0.0%)		
No	43 (100.0%)	7 (100.0%)	6 (100.0%)		
Outcome Variables					
Hospital Length of Stay (days)^a^	7.95 (12.88)	10.29 (4.536)	7.00 (6.89)	.867^c^	0.143^c^
Readmission^b^				.436^d^	1.660^d^
Yes	5 (11.6%)	0 (0.0%)	0 (0.0%)		
No	38 (88.4%)	7 (100.0%)	6 (100.0%)		
Readmission [PE1] Length of Stay (days)^a^	5.61 (2.30)	0 (0.0)	0 (0.0)	--^c^	NA^c^
Unplanned Readmission to Hospital^b^				.436^d^	1.660^d^
Yes	5 (11.6%)	0 (0.0%)	0 (0.0%)		
No	38 (88.4%)	7 (100.0%)	6 (100.0%)		
Mortality^b^				.521^d^	1.302^d^
Yes	4 (9.3%)	0 (0.0%)	0 (0.0%)		
No	39 (90.7%)	7 (100.0%)	6 (100.0%)		

**Figure 2:**
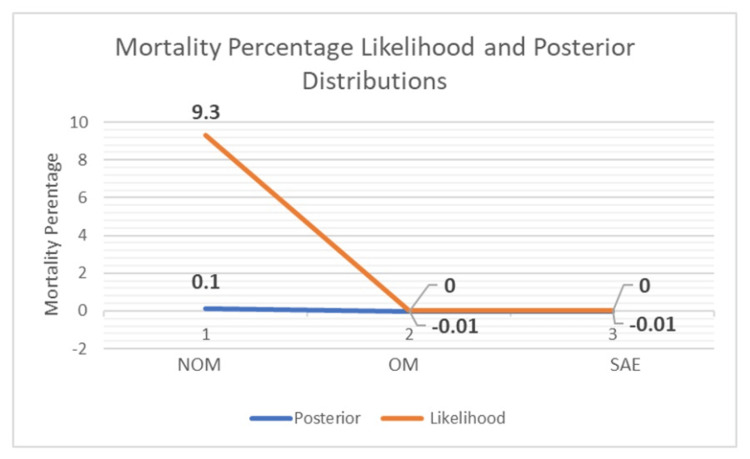
Mortality Proportion Likelihood and Posterior Distributions NOM: non-operative management; OM: operative management; SAE: splenic angioembolization.

**Figure 3:**
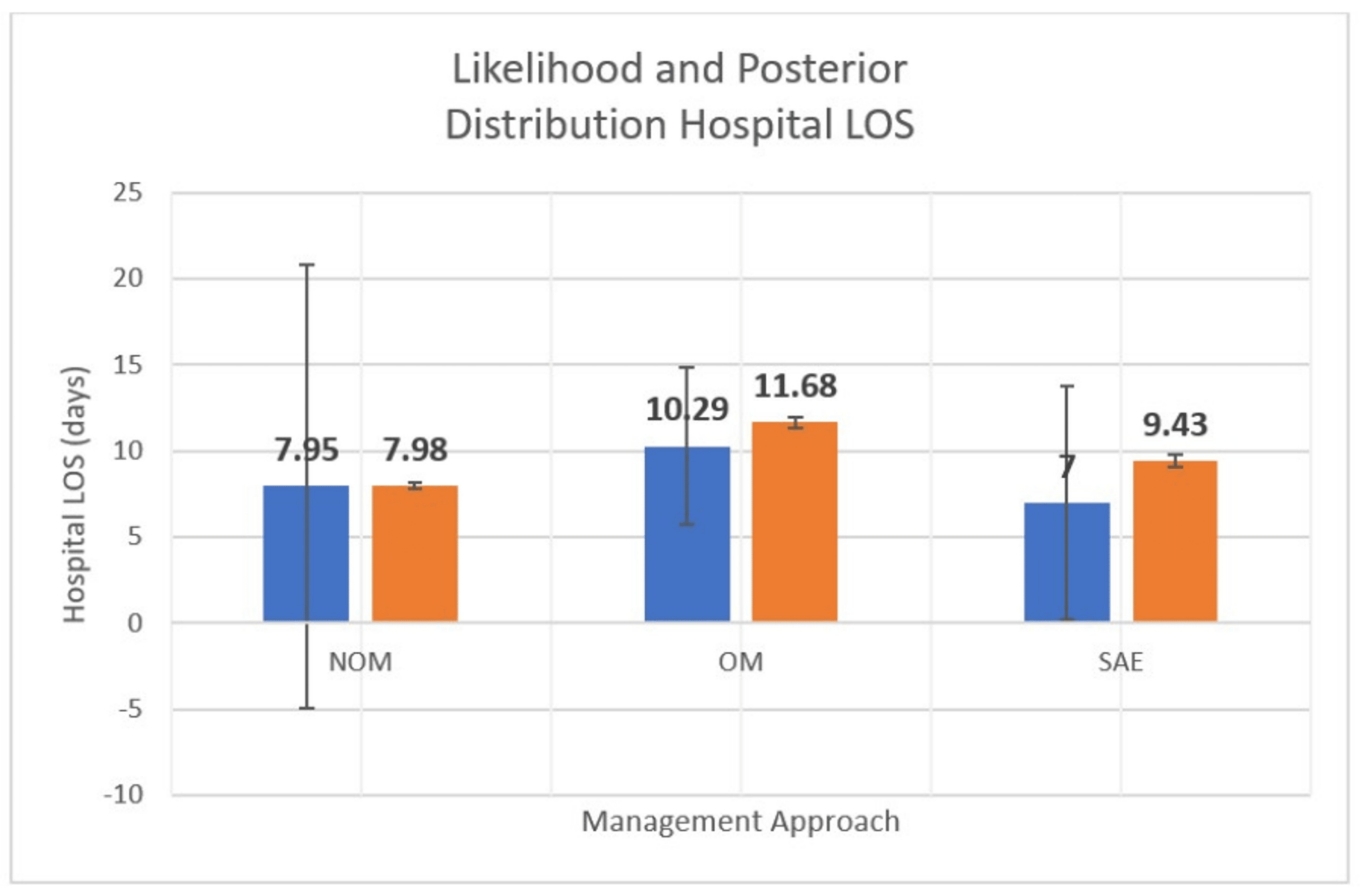
Likelihood and Posterior Distributions Hospital LOS (Mean, SD) LOS: length of stay (in days); SD: standard deviation; NOM: non-operative management; OM: operative management; SAE: splenic angioembolization.

Further comparisons between OM and SAE on the primary outcome of interest - mortality - found that there was no difference with 0% in each group. Additionally, LOS was not significantly different (10.8 (4.7), and 4.6 (1.8) P = .86) between the two groups (Table 3). Bayesian 95% highest density interval (HDI) estimates of mortality in the posterior distribution ([0.02; 0.18], [-6.4e-23; 0.3], and [2.2e-22; 0.3]) and hospital mean ([7.7; 8.3], [11.0; 12.3], and [8.7; 10.2]) (Table 4) provided reduced uncertainty in point and dispersion estimates (Figures 2, 3).

**Table 3:** Comparison of Variables between Surgery and Angioembolization (n = 13) ^a^Mean, SD. ^b^Frequency, percentage. ^c^One-way analysis of variance estimates (ANOVA) (f statistic) were conducted for continuous variable. ^d^Crosstabulation chi-square estimates were conducted for categorical data (except when expected count < 5 in a cell Fisher’s exact statistic was estimated). ^e^Inferential statistic calculated. *Significant p value <.05. ED: emergency department; ISS: injury severity score; RR: respiratory rate; BMI: basal metabolic index; INR: international normalized ratio; LOS: length of stay; ICU: intensive care unit; PRBC: packed red blood cells.

Characteristics	Surgery (n=7)	Angioembolization (n=6)	p value^c,d^	Test statistics^c,d^
Age^a^	43.3 (18.84)	52.20 (19.48)	.447^c^	0.788^c^
Sex^b^			.612^d^	0.258^d^
Male	5 (71.4%)	5 (83.3%)		
Female	2 (28.6%)	1 (16.7%)		
Race^b^			.503^d^	1.376^d^
White	4 (57.1%)	5 (83.3%)		
Black	1 (14.3%)	0 (0.0%)		
Other	2 (28.6%)	1 (16.7%)		
Ethnicity Hispanic/Latino^b^			.261^d^	1.264^d^
Yes	0 (0.0%)	1 (16.7%)		
No	7 (100.0%)	5 (83.3%)		
Extrication^b^			--^d^	NA^d^
Yes	0 (0.0%)	0 (0.0%)		
No	7 (100.0%)	6 (100.0%)		
Time from Injury – Arrival^b^			.446^d^	2.667^d^
< 30 minutes	2 (33.3%)	0 (0.0%)		
30-60 minutes	2 (33.3%)	2 (33.3%)		
61-90 minutes	0 (0.0%)	0 (0.0%)		
91 minutes-12 hours	1 (16.7%)	2 (33.3%)		
>12 hours	1 (16.7%)	2 (33.3%)		
Fall^b^			.308^d^	1.040^d^
Yes	4 (57.15)	5 (83.3%)		
No	3 (42.9%)	1 (16.7%)		
Fall distance in feet^a^	0.1 (0.05)	2.25 (2.87)	.356^c^	1.042^c^
Spleen Grade^a^	3.43 (1.29)	3.0 (1.09)	.505^c^	0.690^c^
Spleen Grade^b^			.692^d^	0.737^d^
Grade 1	0 (0.0%)	0 (0.0%)		
Grade 2	1 (14.3%)	2 (33.3%)		
Grade 3	4 (57.1%)	3 (50.0%)		
Grade 4	0 (0.0%)	0 (0.0%)		
Grade 5	2 (28.6%)	1 (16.7%)		
ED Revised Trauma Score^a^	7.44 (0.64)	7.70 (0.33)	.445^c^	0.799^c^
ED Arrival Injury Severity Score (ISS)^a^	11.50 (3.11)	14.50 (6.28)	.451^c^	0.781^c^
ED Arrival Temperature (F)^a^	98.0 (0.01)	98.0 (1.22)	.820^c^	0.234^c^
ED Arrival Heart Rate^a^	102.75 (27.37)	97.0 (12.68)	.704^c^	0.390^c^
ED Arrival Respiratory Rate (RR)^a^	18.50 (1.29)	18 (3.08)	.273^c^	1.168^c^
ED Respiratory Rate Assisted^b^			.261^d^	1.264^d^
Yes	0 (0.0%)	1 (16.7%)		
No	7 (100.0%)	5 (83.3%)		
ED Arrival Systolic Blood Pressure^a^	113.57 (16.98)	105.67 (12.01)	.632^c^	0.952^c^
Basal Metabolic Index (BMI)^a^	23.41 (3.73)	26.21 (4.02)	.012*^c^	3.053^c^
1^st^ Hematocrit^a^	31.76 (9.71)	39.05 (2.89)	.199^c^	1.417^c^
1^st^ INR^a^	1.10 (0.08)	1.00 (0.01)	.117^c^	1.890^c^
ED Shock Index^a^	0.90 (0.23)	0.92 (0.14)	.818^c^	0.235^c^
ED Reverse Shock Index^a^	1.16 (0.26)	1.10 (0.15)	.605^c^	0.533^c^
ED High Shock Index^b^			.797^d^	0.066^d^
Yes	3 (42.9%)	3 (50%)		
No	4 (57.1%)	3 (50%)		
Number of Comorbidities^a^	0.67 (1.03)	2.20 (1.48)	.043*^c^	2.293^c^
ED LOS (hours)^a^	2.95 (1.76)	3.91 (3.25)	.515^c^	0.673^c^
ED Disposition^b^			.298^d^	2.423^d^
Floor	0 (0.0%)	0 (0.0%)		
Observation	0 (0.0%)	1 (16.7%)		
Step Down	0 (0.0%)	0 (0.0%)		
Intensive Care Unit	2 (28.6%)	3 (50.0%)		
Operating Room	5 (71.4%)	2 (33.3%)		
ICU Admission During Hospital Admission^b^			--^d^	NA^d^
Yes	7 (100.0%)	6 (100.0%)		
No	0 (0.0%)	0 (0.0%)		
ICU LOS (days)^a^	5.71 (3.15)	5.0 (5.44)	.773^c^	0.296^c^
Ventilator during Hospital Admission^b^			.067^d^	3.343^d^
Yes	3 (42.9%)	0 (0.0%)		
No	4 (57.1%)	6 (100.0%)		
Admit Service^b^			.261^d^	1.264^d^
Trauma	7 (100.0%)	5 (83.3%)		
Internal Medicine	0 (0.0%)	0 (0.0%)		
Cardiology	0 (0.0%)	1 (16.7%)		
PRBC (0-24 hrs)^b^			.045*^d^	5.600^d^
Yes	7 (100.0%)	2 (40.0%)		
No	0 (0.0%)	3 (60.0%)		
Plasma (0-24 hrs)^b^			.192^d^	2.343^d^
Yes	3 (42.9%)	0 (0.0%)		
No	4 (57.1%)	6 (100.0%)		
Platelets (0-24 hrs)^b^			.026*^d^	4.952^d^
Yes	4 (57.1%)	0 (0.0%)		
No	3 (42.9%)	6 (100.0%)		
Cryo (0-24 hrs)^b^			.269^d^	NA^d^
Yes	2 (28.6%)	0 (0.0%)		
No	5 (71.4%)	6 (100.0%)		
VLLA (0-24 hrs)^b^			--^d^	NA^d^
Yes	0 (0.0%)	0 (0.0%)		
No	7 (100.0%)	6 (100.0%)		
Cellsaver (0-24 hrs)^b^			--^d^	NA^d^
Yes	0 (0.0%)	0 (0.0%)		
No	7 (100.0%)	6 (100.0%)		
Hospital Length of Stay (days)^a^	10.29 (4.536)	7.00 (6.89)	.337^c^	1.009^c^
Readmission^b^			--^d^	NA^d^
Yes	0 (0.0%)	0 (0.0%)		
No	7 (100.0%)	6 (100.0%)		
Readmission Length of Stay (days)^a^	--	--	--^c^	--^c^
Unplanned Readmission to Hospital^b^			--^d^	NA^d^
Yes	0 (0.0%)	0 (0.0%)		
No	7 (100.0%)	6 (100.0%)		
Mortality^b^			--^d^	NA^d^
Yes	0 (0.0%)	0 (0.0%)		
No	7 (100.0%)	6 (100.0%)		

**Table 4:** Comparison of Estimates of LOS and Mortality using Frequentist and Bayesian Approaches ^a^Mean, SD. ^b^Frequency, percentage. ^c^Independent sample t-tests were conducted for continuous variable estimates. ​​​​​​​^d^Crosstabulation chi-square estimates were conducted for categorical data (except when expected count < 5 in a cell Fisher’s exact statistic was estimated). LOS: length of stay; NOM: non-operative management; AE: angioembolization; HDI: highest density interval.

	Frequentist					Bayesian		
	NOM (n=43)	Surgery (n=7)	AE (n=6)	P value^c,d^	Test statistics^c,d^	NOM (n=85,793)	Surgery (n=21,999)	AE (n=3,895)
Hospital Length of Stay (days)	--	--	--	--	--	--	--	--
Prior Distribution	NA	NA	NA	NA	--	7.56 (10.37)	13.0 (14.87)	11.94 (14.3)
Likelihood Distribution^ a^	7.95 (12.88)	10.29 (4.536)	7.00 (6.89)	.867^c^	0.143^c^	7.95 (12.88)	10.29 (4.536)	7.00 (6.89)
Posterior Distribution	NA	NA	NA	NA	--	7.98 (0.15)	11.68 (0.332)	9.43 (0.377)
HDI	NA	NA	NA	NA	--	7.7; 8.3	11.0; 12.4	8.7; 10.2
Mortality	--	--	--	--	--	--	--	--
Prior Distribution (Beta)	NA	NA	NA	NA	--	Be (0.11,89)	Be (0.1,6.9)	Be (0.1,8.8)
Yes	NA	NA	NA	NA	--	3053 (3.7%)	2,503 (11.5%)	185 (4.9%)
No	NA	NA	NA	NA	--	82,740 (96.3%)	19,496 (88.5%)	3710 (95.1%)
Likelihood Distribution^b^	--	--	--	.52^d^	--^d^	--	--	--
Yes	4 (9.3%)	0 (0.0%)	0 (0.0%)	--	--	4 (9.3%)	0 (0.0%)	0 (0.0%)
No	39 (90.7%)	7 (100.0%)	6 (100.0%)	--	--	39 (90.7%)	7 (100.0%)	6 (100.0%)
Posterior Distribution (Beta)	NA	NA	NA	NA	--	Be (4.1, 39.9)	Be (0.1, 7.9)	Be (0.1, 6.9)
HDI	NA	NA	NA	NA	--	0.019; 0.18	-6.4^e-23^; 0.28	-2.2^e-22^; 0.3

## Discussion

The frequentist approach to the estimates of differences between BSI management and mortality and mean LOS outcomes was limited by a small sample size for inferential estimates to be appropriately powered. However, using a Bayesian approach for analyses provided prior evidence from a large nationally representative study of mortality and mean LOS between approaches to BSI treatment that could inform the frequentist “likelihood” sample. The Bayesian estimates also provide an intuitive interpretation of interval estimates fixed ranges, such as an HDI to which a parameter is known to belong with a prespecified probability. The HDIs estimated for posterior distributions provide a plausible range of values for a parameter that has incorporated findings from additional studies.

Existing literature cites NOM as the standard of care in hemodynamically stable BSI patients [19,20]. There is a statistically high success rate associated with this approach and it can minimize the obligation of choosing more invasive methods with their subsequent complication risks [21,22]. Clinically, the results of our study support this current standard approach. As seen in Table 2, variables including time from injury to ED arrival, splenic injury grade, systolic BP upon arrival to ED, ED Reverse Shock Index, ED High Shock Index, ED LOS, and ED disposition were all found to be significant predictors of hospital LOS and hospital mortality in management of BSI with NOM, OM and SAE treatment options. These findings are in alignment with prior studies that have shown statistically significant differences among values of blood pressure, heart rate, and shock index observed in patients treated with NOM as compared to those treated with splenectomy [1]. Although there was no significant difference in hospital mortality and LOS between NOM, SAE, and OM found in this study, the benefits of less invasive interventions should be considered. NOM of BSI has shown to lower hospital cost, allow for earlier discharge, prevent nontherapeutic celiotomies, minimize the risk of intra-abdominal complications, and lead to reduced transfusion rates associated with an overall improvement in mortality of these injuries [20]. Of note, although NOM is the standard of care with innumerable benefits, it should only be considered in environments that have capabilities for close monitoring and clinical evaluations, with an operating room readily available for urgent laparotomy if needed [20,21]. Without these resources, NOM may not be the safest option for the patient, and alternatives, such as SAE, must be considered. The consideration for missed injuries when selecting NOM as a management technique is critical to keep in mind as well [23]. Moreover, it is important to note that the availability of resources and timeliness play vital roles when considering additional BSI management approaches. A 2020 multicenter study examining the timing of SAE for hemorrhagic control in blunt trauma patients found significantly increased 24-hour mortality when angioembolization was delayed [24]. Factors such as the availability of resources and interventional radiology readiness are consequential when choosing alternatives if NOM is not the most appropriate option. Overall, conservative NOM management of BSI represents a feasible and safe treatment option in many cases, however, the choice of treatment ultimately must be evaluated on a case-by-case basis considering all-encompassing factors [1,21].

Limitations

This study has some limitations. The initial smaller sample size from this single-site study (n=56) leads to increased probability of a type 1 error due to decreased power. Additionally, the single-site sample included data from when the institution was designated a level II trauma center, transitioning to a level I center. The assumption that the resources available during this time for BSI management were consistent with a level I center as opposed to a level II needs to be considered when interpreting these results. Further, the Bayesian analysis sample also examined data from level 1 trauma centers; and the access to resources and increased capabilities at level 1 trauma centers could yield different outcomes with BSI as compared to lower-level trauma centers [7]. Temporal differences between the two samples are also a limitation of the study. The sample used in the Bayesian analysis was from 2007-2015 (prior to Coronavirus-19 [COVID-19]) and the single-site study used sampling from 2021-2022, post-COVID-19. These differences in the sampling timeline and the impact of COVID-19 may alter mortality and LOS outcome estimates when comparing the samples. It has been well established that the COVID-19 pandemic severely disrupted healthcare across the globe as resources were reallocated to COVID-19 services and capacity for healthcare access and treatment was often suboptimal [25, 26]. Additionally, specific to trauma patients such as BSI patients during this period, many trauma centers adopted restrictive transfusion protocols and modified their massive transfusion protocols in response to guidance from blood supply providers anticipating a national shortage of blood products [27]. Though historical threats to external validity, such as the pandemic, cannot be controlled for, as the measurement and handling of the COVID-19 hypothesized confounding variables for trauma patient outcomes are not yet well defined, the consideration of the limitation in inferential discussion must be included [28]. Lastly, the 2007-2015 window seen in the Bayesian analysis must also be considered; the extended time range and resulting medical advancements yield differences in surgical technique, physician expertise with SAE, medications, and treatment, which may also impact the outcomes observed in this study. Despite these limitations, the results still support the current literature and current approaches to BSI management.

## Conclusions

The purpose of this study was to examine mortality and LOS associated with BSI management techniques using both frequentist and Bayesian methods. Using a frequentist approach, no significant difference was found between management techniques in either outcome variable examined. Utilization of a Bayesian approach for analyses helped to inform the frequentist “likelihood” sample using comparable variables from a larger, representative sample. In addition, the Bayesian approach offers an intuitive interpretation of interval estimates fixed ranges that provide a plausible range of values for a parameter that has incorporated findings from additional studies. The inclusion of findings from large high-quality studies provides increased certainty in estimates from smaller studies. Posterior estimates can inform predictive models for testing in future studies. Furthermore, members of this pilot study plan to replicate this study’s design on a larger scale using a national dataset.

## References

[1] Fransvea P, Costa G, Massa G, Frezza B, Mercantini P, BaIducci G (2019). Non-operative management of blunt splenic injury: is it really so extensively feasible? A critical appraisal of a single-center experience. Pan Afr Med J.

[2] Schneider AB, Gallaher J, Raff L, Purcell LN, Reid T, Charles A (2021). Splenic preservation after isolated splenic blunt trauma: the angioembolization paradox. Surgery.

[3] Marsh D, Day M, Gupta A, Huang EC, Hou W, Vosswinkel JA, Jawa RS (2021). Trends in blunt splenic injury management: the rise of splenic artery embolization. J Surg Res.

[4] Harfouche MN, Dhillon NK, Feliciano DV (2022). Update on nonoperative management of the injured spleen. Am Surg.

[5] Sclafani SJ, Shaftan GW, Scalea TM (1995). Nonoperative salvage of computed tomography-diagnosed splenic injuries: utilization of angiography for triage and embolization for hemostasis. J Trauma.

[6] Atkins K, Schneider A, Charles A (2023). Splenic salvage: is there a role for splenorrhaphy in the management of adult splenic trauma?. Am Surg.

[7] Harbrecht BG, Zenati MS, Ochoa JB, Townsend RN, Puyana JC, Wilson MA, Peitzman AB (2004). Management of adult blunt splenic injuries: comparison between level I and level II trauma centers. J Am Coll Surg.

[8] Coccolini F, Montori G, Catena F (2017). Splenic trauma: WSES classification and guidelines for adult and pediatric patients. World J Emerg Surg.

[9] Corn S, Reyes J, Helmer SD, Haan JM (2019). Outcomes following blunt traumatic splenic injury treated with conservative or operative management. Kans J Med.

[10] Winkler RL, Smith JE, Fryback DG (2002). The role of informative priors in zero-numerator problems: being conservative versus being candid. Am Stat.

[11] Baig SA (2022). Bayesian inference: parameter estimation for inference in small samples. Nicotine Tob Res.

[12] Wagenmakers EJ, Marsman M, Jamil T (2018). Bayesian inference for psychology. Part I: Theoretical advantages and practical ramifications. Psychon Bull Rev.

[13] Willan AR, Thabane L (2020). Bayesian methods for pilot studies. Clin Trials.

[14] Brard C, Hampson LV, Gaspar N, Le Deley MC, Le Teuff G (2019). Incorporating individual historical controls and aggregate treatment effect estimates into a Bayesian survival trial: a simulation study. BMC Med Res Methodol.

[15] Goligher EC, Heath A, Harhay MO (2024). Bayesian statistics for clinical research. Lancet.

[16] Bittl JA, He Y (2017). Bayesian analysis: a practical approach to interpret clinical trials and create clinical practice guidelines. Circ Cardiovasc Qual Outcomes.

[17] Gopinath R (2023). To reveal or to conceal: appropriate statistical analysis is a moral obligation for authors in modern medicine. Indian J Anaesth.

[18] Banbeta A, van Rosmalen J, Dejardin D, Lesaffre E (2019). Modified power prior with multiple historical trials for binary endpoints. Stat Med.

[19] Crichton JC, Naidoo K, Yet B, Brundage SI, Perkins Z (2017). The role of splenic angioembolization as an adjunct to nonoperative management of blunt splenic injuries: a systematic review and meta-analysis. J Trauma Acute Care Surg.

[20] Stassen NA, Bhullar I, Cheng JD (2012). Selective nonoperative management of blunt splenic injury: an Eastern Association for the Surgery of Trauma practice management guideline. J Trauma Acute Care Surg.

[21] Kanlerd A, Auksornchart K, Boonyasatid P (2022). Non-operative management for abdominal solidorgan injuries: a literature review. Chin J Traumatol.

[22] Brillantino A, Iacobellis F, Robustelli U (2016). Non operative management of blunt splenic trauma: a prospective evaluation of a standardized treatment protocol. Eur J Trauma Emerg Surg.

[23] Miller PR, Croce MA, Bee TK, Malhotra AK, Fabian TC (2002). Associated injuries in blunt solid organ trauma: implications for missed injury in nonoperative management. J Trauma.

[24] Chehab M, Afaneh A, Bible L (2020). Angioembolization in intra-abdominal solid organ injury: does delay in angioembolization affect outcomes?. J Trauma Acute Care Surg.

[25] Coleman MP, Forman D, Bryant H (2011). Cancer survival in Australia, Canada, Denmark, Norway, Sweden, and the UK, 1995-2007 (the International Cancer Benchmarking Partnership): an analysis of population-based cancer registry data. Lancet.

[26] Stannard R, Lambert PC, Lyratzopoulos G, Andersson TM, Khan S, Rutherford MJ (2024). The long-lasting impacts of the COVID-19 pandemic on population-based cancer survival: what are the implications for data analysis?. Br J Cancer.

[27] Haut ER, Leeds IL, Livingston DH (2020). The effect on trauma care secondary to the COVID-19 pandemic: collateral damage from diversion of resources. Ann Surg.

[28] Butler AM, Burcu M, Christian JB (2023). Noninterventional studies in the COVID-19 era: methodological considerations for study design and analysis. J Clin Epidemiol.

